# A Manual of Military Surgery; or, Hints on the Emergencies of Field, Camp, and Hospital Practice

**Published:** 1861-07

**Authors:** 


ART. IX.—*A Manual of Military Surgery; or, Hints on the Emergencies of Field, Camp, and Hospital Practice.* By S. D. GROSS, M.D. 18mo., pp. 186. J. B. Lippincott & Co.: Philadelphia, 1861.

THE following extract from the preface of this work will inform the reader of the motives which induced the author to prepare it for the press:—

"The sole object which prompts me to publish this little book is an ardent desire to be useful to the young physicians who have so hurriedly entered the volunteer service, perhaps not always with a full knowledge of the weighty responsibilities of their position. It treats, very succinctly, of various matters not generally discussed, except in large and ponderous volumes, inaccessible in the camp and on the battle-field. It is essentially a book for emergencies; portable, easy of reference, always at hand. The substance of it was originally intended as an article for the July number of the *North American Medico-Chirurgical Review*, and it was not until I had made considerable progress in its composition that the idea suggested itself to my mind that it might, if published separately, be of service to a part of my profession at this particular juncture in our public affairs."

The book consists of thirteen chapters, and of an appendix comprising an account of the regulations for the admission of physicians into the medical staff of the army. The principal topics are distributed in the following order:—

"I. Historical Sketch of Military Surgery. II. Importance of Military Surgery. III. Qualifications and Duties of Military Surgeons. IV. Medical Equipments, Stores, and Hospitals. V. Wounds and other Injuries. VI. Amputations and Resections. VII. Ill Consequences of Wounds and Operations. VIII. Injuries of the Head, Chest, and Abdomen. IX. Diseases Incident to

Troops. X. Military Hygiene. XI. Disqualifying Diseases. XII. Feigned Diseases. XIII. Medical, Surgical, and Dietetic Formulæ."

Without making any pretensions to a treatise on military surgery, this little volume comprises, within the smallest possible limits, really all the leading facts, with many important statistics of the subject, and we have no hesitation in recommending it as a safe and useful guide to the young army and naval surgeon. Hospital stewards and nurses, too, will find it of great value in the discharge of their important duties. Every page abounds in practical precepts.

